# Prevalence of depression and anxiety among individuals with preserved ratio impaired spirometry: a systematic review and meta-analysis

**DOI:** 10.3389/fmed.2026.1888761

**Published:** 2026-07-30

**Authors:** Lazzat Zhamaliyeva, Nurlan Brimkulov, Aiganysh Mamurzhankyzy

**Affiliations:** 1 Department of General Practice No. 2, Marat Ospanov West Kazakhstan Medical University, Aktobe, Kazakhstan; 2 Department of Family Medicine, I.K. Akhunbaev Kyrgyz State Medical Academy, Bishkek, Kyrgyzstan

**Keywords:** anxiety, depression, mental health, prevalence, PRISm, spirometry

## Abstract

At present, the preserved ratio impaired spirometry (PRISm) phenotype is attracting the attention of researchers as a new type of lung function impairment. Although its association with cardiovascular and metabolic diseases has been well studied, its link with mental disorders is not fully understood. Therefore, it is necessary to systematically assess the prevalence of depression and anxiety in people with PRISm. This systematic review and meta-analysis was registered in the PROSPERO database (CRD420261353805). Literature was searched in the Scopus and Web of Science, PubMed databases. Cross-sectional or cohort studies were included if the PRISm phenotype was defined by FEV1/FVC ≥ 0.70 and FEV1 < 80% predicted, and if the prevalence of depression or anxiety was reported in numerical form. Data were extracted by two independent reviewers, and pooled prevalence was calculated using a random-effects model. A total of 6 studies (*N* = 43,900) were included in the meta-analysis. The pooled prevalence of depression among PRISm participants was 0.17 (95% CI: 0.11–0.25). In addition, based on 5 studies (*N* = 42,337), the prevalence of anxiety was 0.15 (95% CI: 0.07–0.32). The prevalence varied across individual studies, and high heterogeneity was observed (I^2^ = 97.8% for depression and I^2^ = 99.5% for anxiety). Depression and anxiety are common in people with the PRISm phenotype. These findings highlight the importance of assessing mental health and early intervention in this group in clinical practice. Because of the limited number of included studies, subgroup analyses and meta-regression could not be performed, and the findings should be interpreted with caution. Future large prospective studies are needed to clarify the causal relationship between PRISm and mental disorders.

## 1 Introduction

Depression and anxiety are among the most common mental disorders in the world and make a significant contribution to reduced work capacity, lower quality of life, and increased mortality (1). According to the WHO, depressive disorders affect approximately 3–5% of the global population, while anxiety disorders affect approximately 3–4% (2, 3). The high socio-economic burden of mental disorders highlights the need for their early detection and prevention.

Chronic lung diseases, especially obstructive lung damage (COPD), have long been known to be closely associated with the development of depression and anxiety. However, in recent years, a new lung function impairment phenotype – preserved ratio impaired spirometry (PRISm) – has attracted the attention of clinical and epidemiological studies. The PRISm phenotype is characterized by a preserved forced expiratory volume in one second to forced vital capacity ratio (FEV1/FVC) and a reduced FEV1 level and is found in 7–20% of the general population (4, 5).

Recent large cohort studies have shown that people with PRISm have higher rates of cardiovascular diseases, metabolic disorders, and overall mortality. In addition, a 2024 study by Yang et al., based on UK Biobank data, found that the PRISm phenotype increases the risk of developing depression and anxiety and is associated with higher mortality related to mental disorders in this group (6). However, studies conducted in different countries report varying prevalence rates of depression among people with PRISm, which highlights the need to combine these data and conduct a pooled quantitative analysis.

To date, no systematic review or meta-analysis has specifically synthesized evidence on the prevalence of depression and anxiety among individuals with PRISm. Therefore, combining the results of different studies and calculating a pooled prevalence can help provide a scientific basis for the need to screen individuals with the PRISm phenotype for mental disorders in clinical practice.

The objective of this systematic review and meta-analysis was to estimate the prevalence of depression and anxiety among individuals with PRISm.

## 2 Materials and methods

### 2.1 Protocol and reporting

This systematic review and meta-analysis was registered in the PROSPERO international database (registration number: CRD420261353805). The protocol can be accessed through the PROSPERO record for this review. No amendments were made to the information provided at the time of registration. The study was conducted in accordance with PRISMA guidelines.

### 2.2 Eligibility criteria

Study selection was carried out based on predefined inclusion and exclusion criteria. Studies were included if the PRISm phenotype was defined using spirometry as FEV1/FVC ≥ 0.70 and FEV1 < 80% predicted. In addition, studies had to report the prevalence of depression and anxiety in people with PRISm in numerical form, either as prevalence or as the number of events. Population-based cross-sectional or cohort observational studies with full text available were included. Studies were accepted if depression and/or anxiety were identified using standard scales, such as the Hospital Anxiety and Depression Scale (HADS), or by clinical diagnosis based on medical records or self-report. Studies were excluded if they were review articles, case reports, animal studies, or did not provide extractable data relevant to the outcomes of interest. When duplicate publications from the same population were identified, only the most complete and latest study was included.

### 2.3 Information sources and search strategy

A systematic literature search was performed in Scopus and Web of Science, PubMed databases. The search covered articles published from 1 January 2019 to 30 March 2026, and the final search of both databases was conducted on 30 March 2026. The search was limited to publications in English. Studies were included if the full text was available and sufficient quantitative data could be extracted for meta-analysis. The search strategy was developed by combining free-text keywords and subject terms. The main search terms used were: (“preserved ratio impaired spirometry” OR “PRISm” OR “COPD” OR “chronic obstructive pulmonary disease”) AND (“depression” OR “anxiety”). Boolean operators were used in the correct combination, and terms describing the PRISm phenotype were combined with terms describing depression and anxiety. An additional manual search was performed by reviewing the reference lists of the included articles allowed the identification of additional relevant studies. The reference lists of the included studies were also checked on 30 March 2026. The titles and abstracts of the identified articles were screened by two independent reviewers, and full-text articles that met the selection criteria were included in the meta-analysis.

### 2.4 Source selection process

**Figure 1:**
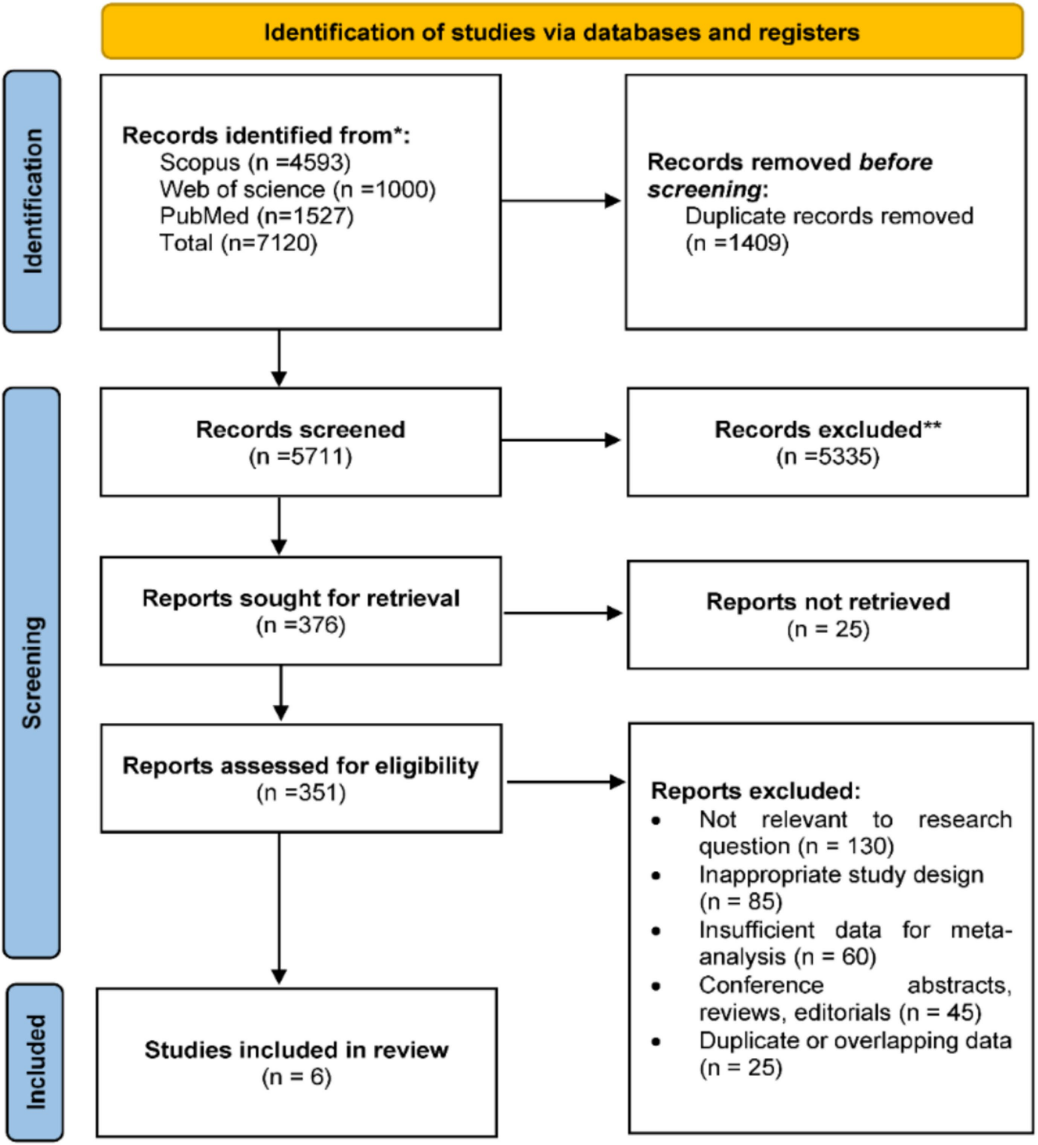
Schematic representation of the study selection process.

All records identified through the literature search were first exported from the databases. Duplicate records were then removed. The titles and abstracts of the remaining records were screened by two independent reviewers. Records that clearly did not meet the eligibility criteria were excluded at this stage. The full texts of potentially eligible articles were then assessed in detail. Studies that met the inclusion criteria were included in the final meta-analysis. When several publications used the same cohort or overlapping participant data, we compared the study population, sample size, assessment period, PRISm definition, and reported mental health outcomes. To avoid double counting, only one study from each overlapping dataset was included. Preference was given to the study with the most complete data for depression and/or anxiety prevalence in the PRISm group, the clearest PRISm definition, and the largest or most recent dataset. The excluded overlapping studies were not included in the pooled analysis because including them could have counted the same participants more than once. Disagreements during the selection process were resolved by discussion. If agreement could not be reached, a third reviewer was involved. The study selection process is shown in Figure 1.

### 2.5 Data extraction

Data were extracted from the studies included in the meta-analysis using a pre-prepared standardized form. From each study, the following information was collected: first author, year of publication, country, study design, number of participants, number of people with the PRISm phenotype, outcome assessed (depression and/or anxiety), method used to assess the outcome, cut-off values where applicable, time point of assessment, number of cases, and prevalence in the PRISm group. When depression or anxiety prevalence was reported as a percentage, the number of cases was calculated by multiplying the percentage by the total number of participants in the PRISm group. All data were extracted independently by two reviewers. The results were then compared. In case of disagreements, the final decision was made with the involvement of a third reviewer.

### 2.6 Risk of bias assessment

The methodological quality of the studies was assessed using the Joanna Briggs Institute (JBI) Critical Appraisal Checklist for Studies Reporting Prevalence Data. Each included study was evaluated by two independent reviewers based on the nine criteria of the JBI checklist (C1–C9). For each criterion, the results were recorded as “Yes” (low risk), “No” (high risk), or “Unclear” (unclear risk). In case of disagreements, consensus was reached with the involvement of a third reviewer. The overall risk of bias for each study was based on the combined assessment of all criteria. The results were visualized using robvis software and presented as a traffic-light plot for each criterion.

### 2.7 Data synthesis and statistical analysis

Studies were grouped according to the reported outcome. Studies reporting depression were included in the depression synthesis, and studies reporting anxiety were included in the anxiety synthesis. If a study reported both outcomes, its results were included in both syntheses. Statistical analyses were performed using R version 4.4.2 with the meta package (metaprop()), and forest plots were generated using RStudio. Logit transformation (PLOGIT) was used to stabilize the variance. Because variation between studies was expected, pooled prevalence estimates were calculated using a random-effects model. Between-study variance (τ^2^) was estimated using the maximum likelihood (ML) method. Heterogeneity was assessed using the Cochran Q test and the I^2^ statistic. I^2^ values greater than 50% were considered to indicate substantial heterogeneity. The prevalence from each study and the pooled result were presented using forest plots, showing the proportions of individual studies, 95% confidence intervals, and the overall pooled prevalence. All results were reported with 95% confidence intervals, and statistical significance was set at *p* < 0.05. A sensitivity analysis using the leave-one-out approach was performed to assess the stability of the pooled prevalence estimates for depression and anxiety. In this analysis, each study was removed in turn, and the pooled estimate was recalculated to assess the influence of individual studies on the overall result.

### 2.8 Publication bias

Assessment of publication bias (e.g., funnel plot and Egger’s test) was not performed due to the small number of included studies, as such methods are not reliable when fewer than 10 studies are available. In addition, subgroup analysis was not performed due to the limited number of included studies.

### 2.9 Patient and public involvement

Patients and/or the public were not involved in the design, conduct, reporting, or dissemination plans of this research, as this study was a systematic review and meta-analysis based on previously published data.

## 3 Results

### 3.1 Search results and study characteristics

Six studies that met the inclusion criteria were included in the meta-analysis (Figure 1). In total, 43,900 participants with the PRISm phenotype were included across these studies. Five studies had a cross-sectional design, while one was a prospective cohort study. The studies were conducted in the USA, South Korea, China, the UK, and Saudi Arabia. The main characteristics of the included studies are presented in Table 1. In all studies, PRISm was defined based on a preserved FEV1/FVC ratio (≥0.70) and reduced FEV1 (<80% predicted). However, there were some differences in how spirometry was assessed. Two studies used post-bronchodilator measurements, while the others used pre-bronchodilator spirometry. The number of PRISm participants ranged from 101 to 38,879 across the studies. Data on depression were available in all six studies, while data on anxiety were reported in five studies. The study populations were not uniform. Two studies included current or former smokers with at least a 10 pack-year history from the COPDGene project. One study included patients attending a pulmonary clinic with respiratory symptoms. The remaining studies were population-based and included middle-aged and older adults from community samples or national surveys. Mental health outcomes were assessed using different methods. Four studies used the Hospital Anxiety and Depression Scale (HADS), while two studies used diagnosis-based methods, including physician diagnosis and/or ICD-10-coded data. These differences may have contributed to the high heterogeneity between studies. Further details are provided in Table 1.

**Table 1:** Characteristics of included studies.

Author (Year)	Country	Study design	Setting/population	PRISm definition	PRISm sample size (*N*)	Outcome measured	Tool/diagnostic method	Cut-off	Timepoint	Events (*n*)
Iyer et al., 2019 (7)	USA	Cross-sectional analysis	Ambulatory current/former smokers (≥10 pack-years) enrolled in COPDGene	Post-bronchodilator: FEV1/FVC ≥ 0.70 and FEV1%pred <0.80	662	Depression Anxiety	HADS (Hospital Anxiety and Depression Scale)	HADS-D ≥ 8; HADS-A ≥ 8	Phase 2 visit (baseline cross-sectional)	Depression: 109 (16.5% of 662, calculated); Anxiety: 147 (22.2% of 662, calculated)
Kim et al., 2022 (8)	South Korea	Cross-sectional population-based study	General population ≥50 years, national health survey (KNHANES 2007–2015)	Pre-bronchodilator: FEV1/FVC ≥ 0.70 and FEV1 < 80% predicted	1,563	Depression	Self-reported physician diagnosis (questionnaire-based comorbidity survey)	Diagnosis-based (no scale)	Baseline survey	234
Koo et al., 2022 (9)	USA	Cross-sectional (10-year follow-up COPDGene)	Current & former smokers ≥10 pack-years	FEV1/FVC ≥ 0.70 and FEV1 < 80% predicted	236	Depression Anxiety	Self-reported doctor diagnosis + HADS questionnaire	HADS-D ≥ 8; HADS-A ≥ 8	10-year follow-up visit	Depression: 76; Anxiety: 58
Lei et al., 2024 (10)	China	Cross-sectional population-based study	General adult population from community health survey	Pre-bronchodilator: FEV1/FVC ≥ 0.70 and FEV1 < 80% predicted	2,459	Depression Anxiety	HADS	HADS-D ≥ 8; HADS-A ≥ 8	Baseline survey	Depression: 266; Anxiety: 208
Yang et al., 2024 (6)	UK/UK Biobank	Prospective cohort (longitudinal; Cox models)	General population, middle-aged/older adults in UK Biobank	Pre-bronchodilator FEV1/FVC ≥ 0.70 and FEV1 < 80% predicted	38,879	Depression Anxiety	Doctor-diagnosed; ICD-10 codes (from health records + self-report)	Diagnosis-based (no questionnaire cut-off)	Baseline (recruitment)	Depression: 3,616 (calculated from 9.3% of 38,879); Anxiety: 1,516 (calculated from 3.9% of 38,879)
Alqarni et al. (2026) (11)	Saudi Arabia	Hospital-based cross-sectional study	Patients attending a pulmonary clinic with respiratory symptoms at King Abdulaziz University Hospital, Jeddah	Post-bronchodilator FEV1/FVC ≥ 0.70 and post bronchodilator FEV1 < 80% predicted	101	Depression Anxiety	HADS (Hospital Anxiety and Depression Scale)	HADS-D ≥ 8; HADS-A ≥ 8	Study visit/cross-sectional assessment, November 2023 to October 2024	Depression: 27; Anxiety: 38

PRISm, Preserved Ratio Impaired Spirometry; FEV1, Forced Expiratory Volume in one second; FVC, Forced Vital Capacity; FEV1/FVC, Ratio of forced expiratory volume in one second to forced vital capacity; HADS, Hospital Anxiety and Depression Scale; HADS-D, Hospital Anxiety and Depression Scale depression subscale; HADS-A, Hospital Anxiety and Depression Scale anxiety subscale; COPD, Chronic Obstructive Pulmonary Disease; KNHANES, Korea National Health and Nutrition Examination Survey; ICD-10, International Classification of Diseases, 10th Revision; GLMM, Generalized Linear Mixed Model; CI, Confidence Interval; *N*, sample size; *n*, number of events (cases); I^2^, measure of heterogeneity in meta-analysis; τ^2^, between-study variance in random-effects mode.

^a^For Iyer et al. (7), event numbers were calculated from the reported prevalence: depression, 662 × 0.165 = 109.23, rounded to 109; anxiety, 662 × 0.222 = 146.96, rounded to 147.

### 3.2 Risk of bias results

**Figure 2:**
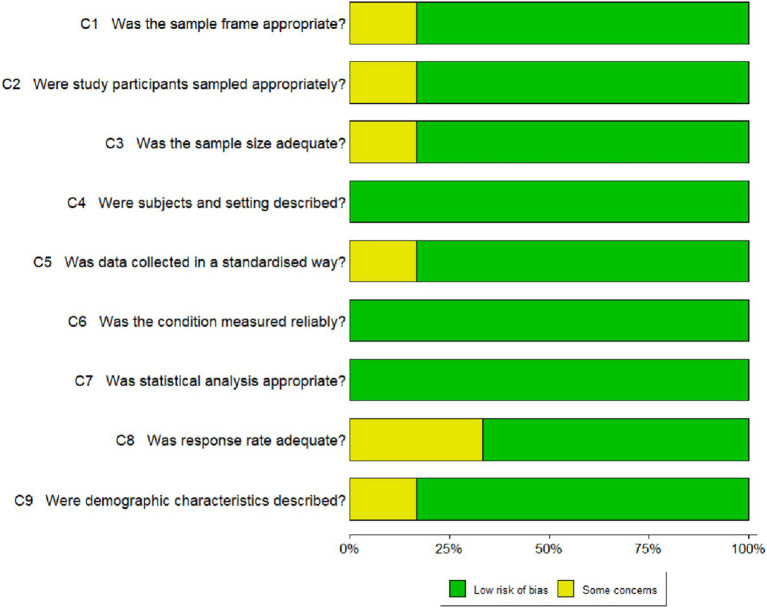
Risk of bias summary plot for included studies using the JBI Critical Appraisal Checklist.

The methodological quality of the six included studies was assessed using the JBI Critical Appraisal Checklist. The risk of bias summary plot showed that most studies had a low risk of bias across the assessed domains. For C4 (subjects and setting description), C6 (reliable measurement of condition), and C7 (appropriate statistical analysis), all studies were rated as low risk. For C1 (sample frame appropriateness), C2 (sampling appropriateness), C3 (sample size adequacy), C5 (standardized data collection), and C9 (demographic characteristics description), five studies were rated as low risk and one study had some concerns. For C8 (response rate adequacy), four studies were rated as low risk, whereas two studies had some concerns because the response rate or the effect of non-response was not clearly described. Overall, the included studies were assessed as having predominantly low risk of bias, with some concerns mainly related to sampling and response rate. The results are shown in Figure 2.

### 3.3 Pooled prevalence of depression among PRISm participants

**Figure 3:**
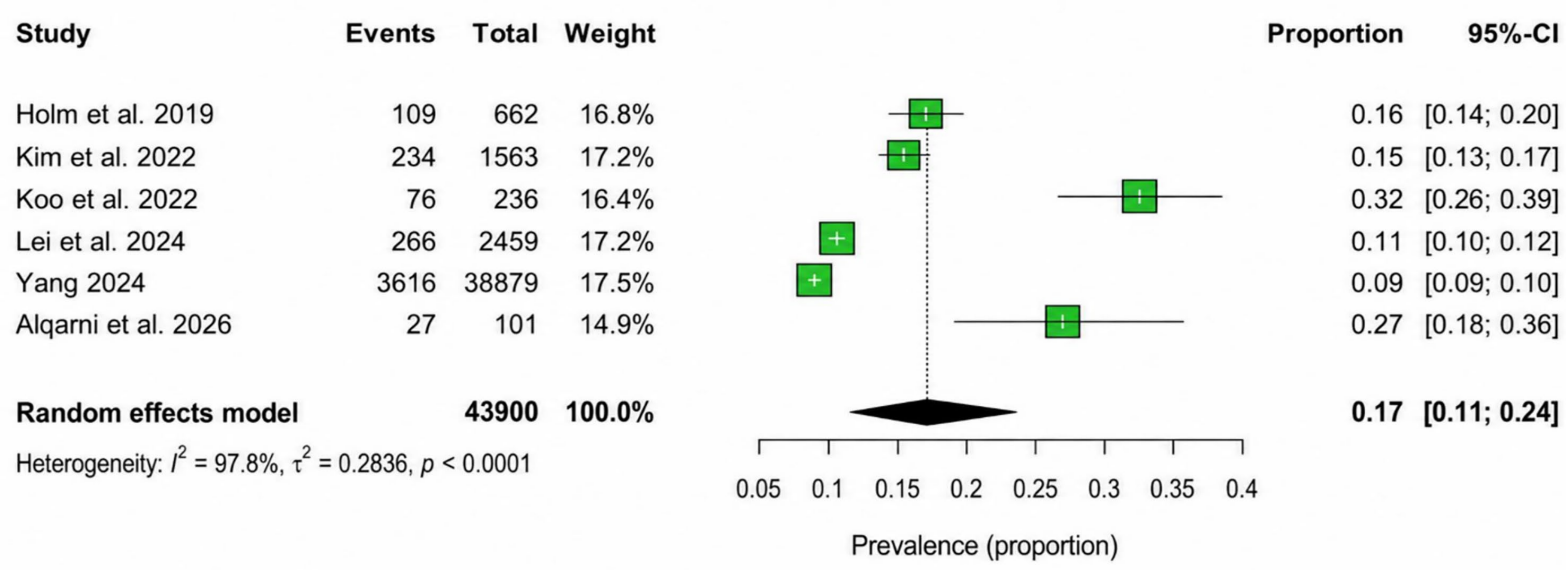
Forest plot of pooled prevalence of depression among PRISm participants.

A total of six studies were included in the meta-analysis, with a total of 43,900 PRISm participants. In each study, prevalence was calculated based on the number of depression cases and the total number of participants in the PRISm group. The meta-analysis using a random-effects model showed that the pooled prevalence of depression among people with the PRISm phenotype was 0.17 (95% CI: 0.11–0.25). The prevalence of depression in individual studies ranged from 0.09 to 0.32. High heterogeneity was observed between studies (I^2^ = 97.8%, τ^2^ = 0.3443, *p* < 0.0001), which can be explained by differences in study populations, measurement methods, and study designs. The results were presented as a forest plot, showing the prevalence of each study, 95% confidence intervals, and the overall pooled prevalence. The results are presented in Figure 3 as a forest plot.

### 3.4 Pooled prevalence of anxiety among PRISm participants

**Figure 4:**
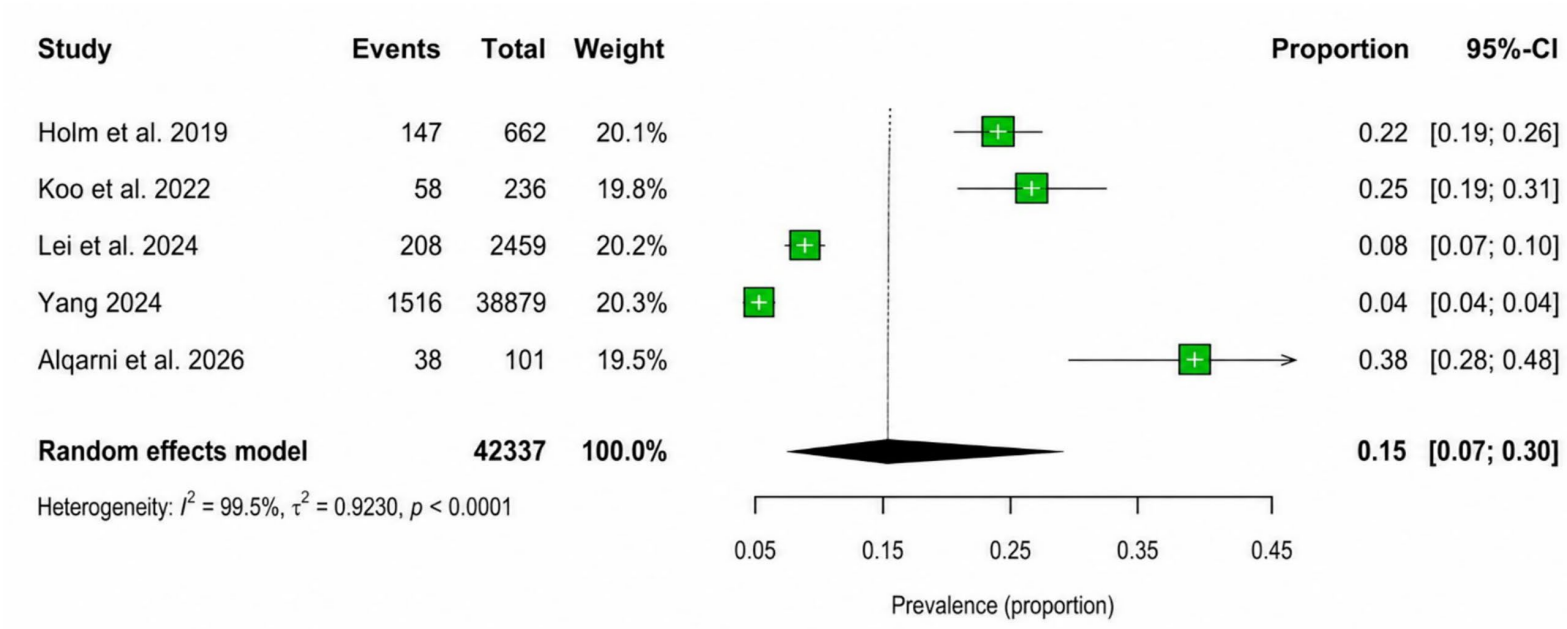
Forest plot of pooled prevalence of anxiety among PRISm participants.

Five studies were included in the meta-analysis to assess the prevalence of anxiety in people with the PRISm phenotype (total *N* = 42,337). The meta-analysis using a random-effects model showed that the pooled prevalence of anxiety was 0.15 (95% CI: 0.07–0.32). The prevalence in individual studies ranged from 0.04 to 0.38. Very high heterogeneity was observed between studies (I^2^ = 99.5%, τ^2^ = 1.1574, *p* < 0.0001), and the results are presented in Figure 4 as a forest plot.

### 3.5 Sensitivity analysis

**Figure 5:**
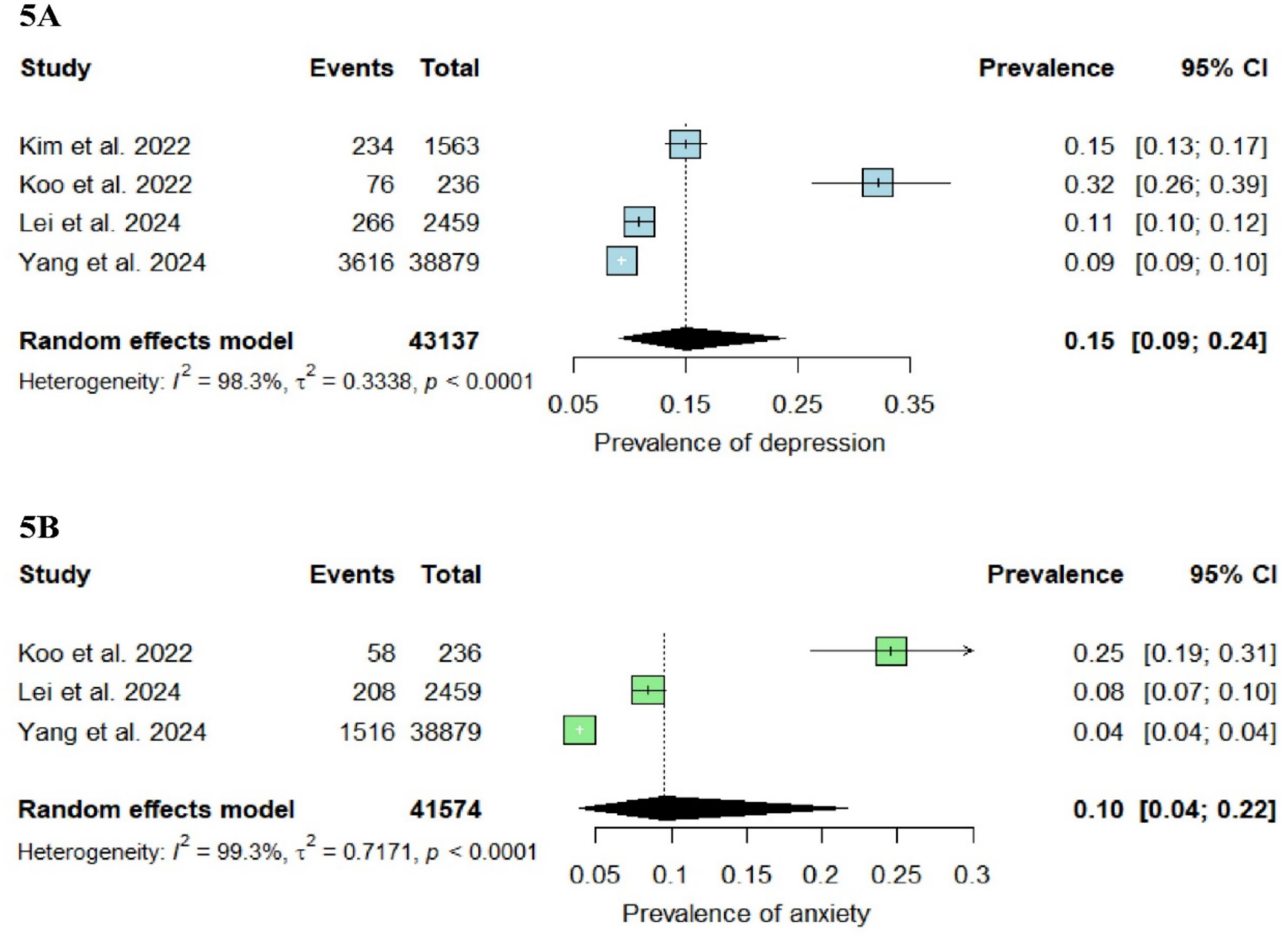
Sensitivity analysis excluding post-bronchodilator studies. **(A)** Depression prevalence. **(B)** Anxiety prevalence.

A sensitivity analysis was performed by excluding studies that used post-bronchodilator spirometry. After excluding these studies, four studies were included in the depression analysis and three studies were included in the anxiety analysis. The pooled prevalence of depression was 0.15 (95% CI: 0.09–0.24), and the pooled prevalence of anxiety was 0.10 (95% CI: 0.04–0.22). Heterogeneity remained high for both outcomes (I^2^ = 98.3% for depression and I^2^ = 99.3% for anxiety). The results are shown in Figure 5.

### 3.6 Certainty of evidence

The certainty of evidence was assessed using the GRADE (Grading of Recommendations Assessment, Development and Evaluation) approach. For both depression and anxiety, the certainty of evidence was rated as very low. This was mainly due to the observational design of the included studies, very high heterogeneity, the small number of studies, and indirectness related to differences in PRISm definitions, study populations, and outcome assessment methods. The GRADE assessment is presented in Table 2.

**Table 2:** Certainty of evidence according to GRADE.

Outcome	Studies	Participants	Pooled prevalence	Certainty of evidence	Main reasons for rating
Depression	6	439	0.17 (95% CI: 0.11–0.25)	low	Observational studies; high heterogeneity; small number of studies; indirectness
Anxiety	5	42,337	0.15 (95% CI: 0.07–0.32)	low	Observational studies; very high heterogeneity; small number of studies; indirectness

## 4 Discussion

This systematic review and meta-analysis assessed the prevalence of depression and anxiety among people with the PRISm phenotype for the first time. By combining the results of six studies, the pooled prevalence of depression among PRISm participants was 0.17 (95% CI: 0.11–0.25). In addition, based on data from five studies, the pooled prevalence of anxiety was 0.15 (95% CI: 0.07–0.32). These findings show that depression and anxiety are common among people with the PRISm phenotype. However, these pooled estimates should be interpreted with caution because of the very high heterogeneity between studies. Also, the small number of included studies may reduce the stability and precision of the pooled prevalence estimates. Therefore, the exact pooled values should be interpreted carefully. Nevertheless, the overall finding suggests that depression and anxiety are common in this population.

The prevalence of depression in individual studies ranged from 0.09 to 0.32 (6–11). For anxiety, the reported values ranged from 0.04 to 0.38 (6, 7, 9–11). These wide differences may be explained by differences in the characteristics of the studied PRISm populations, the countries where the studies were conducted, the study designs, and the methods used to assess mental disorders. In addition, the study by Alqarni and colleagues reported relatively high prevalence of both depression and anxiety. This may have contributed to the increased variation between studies.

Differences in the methods used to define depression and anxiety may also explain part of the heterogeneity. Some studies used the HADS questionnaire, with cut-off values of HADS-D ≥ 8 for depression and HADS-A ≥ 8 for anxiety. This approach allows the identification of clinically important symptoms based on questionnaire responses. Other studies used diagnosis-based definitions, such as physician diagnosis, medical records, or ICD-10 codes. These methods identify formally diagnosed cases rather than symptom severity. Therefore, studies using symptom scales may include a broader group of participants with symptoms of depression or anxiety, whereas diagnosis-based methods may identify only clinically confirmed cases. These differences should be considered when interpreting the pooled prevalence estimates.

Very high heterogeneity was observed for both depression and anxiety in this meta-analysis. Because of the small number of included studies, subgroup analyses and meta-regression could not be performed. Stratifying studies according to diagnostic methods, spirometry type, or geographic region would have resulted in very small subgroups and statistically unstable estimates. Therefore, the sources of heterogeneity could not be explored quantitatively. In addition, this should be considered one of the major limitations of this meta-analysis. Nevertheless, the random-effects model allowed us to account for differences between studies, and the pooled estimates are considered suitable for describing the overall trend. Furthermore, the heterogeneity may reflect clinical differences between population-based cohorts and hospital-based samples, as well as differences between pre-bronchodilator and post-bronchodilator spirometry measurements.

From a clinical perspective, these findings are important. Although people with the PRISm phenotype have impaired lung function, they do not fully meet the classic diagnostic criteria for COPD. Therefore, this group has long been considered an “intermediate phenotype.” However, recent studies have shown that people with the PRISm phenotype have a high burden of cardiovascular and metabolic comorbidities (6). Our findings also showed that mental disorders are common in this group, supporting the need for routine screening for depression and anxiety among patients with the PRISm phenotype.

This meta-analysis has several limitations. First, most of the included studies had a cross-sectional design; therefore, causal relationships cannot be determined. Second, the methods used to assess mental disorders were not consistent, which may lead to measurement bias. Third, the high heterogeneity may limit the precision of the pooled estimates. In addition, because of the small number of studies, subgroup analyses and meta-regression could not be performed to quantitatively explore the possible sources of heterogeneity. A formal assessment of the certainty of evidence was not conducted, and this should be considered when interpreting the findings.

There were also some additional limitations related to the review process itself. The literature search was limited to three databases and publications in English, which may have resulted in the omission of some relevant studies. In addition, the absence of unpublished data does not completely rule out the possibility of publication bias. Although funnel plot analysis and Egger’s test were not performed because of the small number of included studies, the possibility of publication bias cannot be excluded.

## 5 Conclusion

In conclusion, this systematic review and meta-analysis showed a high prevalence of depression and anxiety in people with the PRISm phenotype. These findings highlight the importance of assessing mental health and early intervention in individuals with PRISm in clinical practice. Future large prospective studies are needed to clarify the causal relationship between the PRISm phenotype and mental disorders.

## Data Availability

The original contributions presented in the study are included in the article/supplementary material, further inquiries can be directed to the corresponding author.
